# Combined impact of lemongrass and spearmint herbs on performance, serum metabolites, liver enzymes, and meat quality of broiler

**DOI:** 10.5455/javar.2022.i640

**Published:** 2022-12-31

**Authors:** Md. Aliar Rahman, Sabiha Sultana, Md. Rahat Ahmad Redoy, Momota Rani Debi, Rakhi Chowdhury, Mohammad Al-Mamun

**Affiliations:** Department of Animal Nutrition, Bangladesh Agricultural University, Mymensingh, Bangladesh; † The two authors contributed equally.

**Keywords:** Broilers, lemongrass-spearmint, liver health, meat quality, performance

## Abstract

**Objectives:**

This study aimed to assess the influence of feeding fresh lemongrass (*Cymbopogon citratus*) or spearmint (*Mentha spicata*) and their combination on performance, serum metabolites, liver enzymes, and meat quality in broilers.

**Materials and Methods:**

A total of 168 day-old Indian River chicks were arbitrarily offered four experimental rations: (i) control ration (CT-R): corn-soya-based ration, (ii) lemongrass ration (LG-R): CT-R + 1.0% DM of lemongrass; (iii) spearmint ration (SM-R): CT-R + 1.0% DM of spearmint; and (iv) lemongrass-spearmint ration (LS-R): CT-R + 0.5% DM from both lemongrass and spearmint. Each ration was given to 42 birds for a duration of 35 days, with 3 replications and 14 birds each.

**Results:**

Elevated body weight gain was observed in LG-R (1,502 gm), LS-R (1,492 gm), and SM-R (1,474 gm) compared to CT-R (1,451 gm) (*p* = 0.078). Herbal rations successfully reduced almost 3%–5% of serum and meat total cholesterol concentrations compared to CT-R. Compared to CT-R, the highest zinc and iron concentrations of serum and meat were measured in LG-R and SM-R, respectively, while both minerals of serum and meat were observed to be better in LS-R (*p* < 0.05). Herbal rations significantly improved serum liver enzyme activity and ameliorated the red color of breast and thigh meat but failed to improve the lightness and yellowness of both types of meat compared to CT-R.

**Conclusions:**

LG-R, SM-R, and LS-R improved bird performance, liver health, and meat color, and lowered serum and meat cholesterol levels. But among them, LS-R efficaciously increased the serum and meat zinc and iron concentrations.

## Introduction

Demand for value-added meat has been promoted by rising per capita income, consumer awareness of purchasing antioxidant-rich, safe meat, and the detrimental impact of antibiotic growth promoters (AGPs) used in animal feed on human health. Therefore, scientists are searching for strategic tools as a substitute for AGPs, which enhance the performance and meat quality of broilers. Natural herbs and their extracts are one of the best tools used in broiler feed to replace AGPs since they are abundant in bioactive components, vitamins, and minerals that accelerate nutrient utilization, productivity, immunity, and meat quality [1–3].

Lemongrass (*Cymbopogon citratus*) and its extract (oil) are popularly used in both human therapies and animal production [4]. Lemongrass has a variety of bioactive components like α-citral, β-citral, and limonene, which show anti-inflammatory, immune-modulatory, antioxidant, and antimicrobial activity [5,6]. Lemongrass oil was the best candidate to replace the AGPs in the broiler industry [4,7]. Including lemongrass meal in broiler rations at different doses led to inconsistent results in terms of production performance [8,9].

Spearmint (*Mentha spicata*) has been utilized for human therapy and applied as a phyto-additive in the poultry industry [10,11]. Spearmint is enriched in bioactive components like carvone, limonene, phenolic acids, flavonoids, and ascorbic acid in the leaves, which express antioxidants, antimicrobials, and anti-inflammatory properties [10,12]. Broilers received spearmint leaf powder with the basal ration, accelerating production performance [11,13].

Moreover, both herbs are abundant in bioactive components that have antioxidant, anti-inflammatory, and antibacterial activities. Besides, lemon grass is dense in zinc, whereas spearmint is rich in iron. However, it is well known that these minerals are essential for optimal broiler performance and meat quality. To the best of our knowledge, no studies have been conducted to evaluate the meat quality of broilers provided by these herbs and their combinations.

So, the present research was designed to assess the influence of fresh lemongrass, spearmint and their combination on production performance, serum lipid and mineral profile, liver enzymatic status, and meat quality of broilers.

## Materials and Methods

### Ethical approval

This research was approved by the Animal Welfare and Ethics Committee, Bangladesh Agricultural University, Mymensingh 2202 (AWEEC/BAU/2022 (65)).

### Experimental site, rations, and design

This existing research was conducted at the poultry rearing unit, the Nutrition Lab, and the Feed Safety and Phyto-Nutrition Lab, Department of Animal Nutrition, Bangladesh Agricultural University, Bangladesh. Initially, 168 birds (Indian River) were purchased from a local CP hatchery with an initial weight of 46.0 ± 2.0 gm and kept at the poultry rearing unit for a 35-day feeding trial. For this trial, the birds were arbitrarily offered 4 experimental rations (42 birds per ration), with 14 birds in each replicate with 3 replications in a completely randomized design. The birds were offered starter and grower rations from 1 to 14 and 15 to 35 days, respectively, having 22.47% and 20.83% CP as well as 2,956 and 3,098 kcal/kg ME, which are taken as control rations (CT-R), while birds were served CT-R with fresh lemongrass ration (LG-R) and spearmint leaves (SM-R) at 1.0% on a DM basis and lemongrass and spearmint leaves [lemongrass-spearmint ration (LS-R)] at 0.5% + 0.5% on a DM basis. On the fifth day of life, birds began to supply approximately 1 mm of mature (60-day-old) lemongrass and spearmint leaves.

### Management of birds

From 1 to 35 days, the birds were raised in a 12-floor cage open-sided house, ensuring more than 0.1 m2 space for each bird and having 4‒5 cm sawdust bedding. Feces were mixed with litter, and litter was timely wilted and replaced in the evening to ensure less bird stress. With ensuring strict biosecurity and regular monitoring, birds were reared in the same environment, while providing *ad libitum* water. The drinkers and feeders were properly cleaned on a routine basis. In the first week, birds were kept under a brooder and maintained at 33°C, and then the temperature was gently reduced to 3°C per week until it was maintained at around 21°C until 35 days. Using the Baby Chicks Ranikhet Disease and Gumboro vaccines, birds were vaccinated against Ranikhet and Gumboro diseases on the 4^th^ and 12^th^ days of life, respectively.

### Sample collection and meat color determination

Daily supplied and refused feed were noted, and feed intake (FI) was measured between the supplied and refused feed. Initial body weight and weekly body weight were recorded, and body weight gain (BWG) was calculated from the difference between the initial and final body weight (FBW). Birds and dead birds were monitored and recorded, while the dead bird’s weight was considered to adjust for FI. The ratio of FI to BWG was taken as the feed conversion ratio (FCR), while the performance efficiency factor (PEF) was measured by adopting the formulas of Martins et al. [14]. After taking the average body weight of each replication and tagging the leg band, 15 birds (5 birds per replication) were slaughtered by the Halal method for blood collection, meat color determination, and carcass evisceration at 35 days. Blood samples were collected using a marked Falcon tube and instantly centrifuged at 3,410 rpm for 15 min, and then transferred to a marked Eppendorf tube using a micropipette and stored at −20°C. After complete skin bleeding, the shank, viscera, head, neck, and giblet (heart, liver, spleen, and gizzard) were separated. Then the dressing yield (DY) was measured after taking the carcass weight, while the weight of the liver and abdominal fat percent was taken and calculated on a percentage basis. After cleaning the birds with fresh water, the breast and thigh were removed, weighed, and the right portion of each part was placed in the refrigerator at 4°C for 24 h. The meat sample was taken out of the freezer and cleaned with a fresh cloth. The calorimeter (CR-410 colorimeter, Minolta, Japan) was inserted into the breast and thigh meat following the manufacturer’s guidelines. Meat color was denoted by lightness (*L*), redness (*a*), and yellowness (*b*), while saturation index (SI) was calculated by the following equation: SI = SI = √(*a*^2^ + *b*^2^).

### Sample analysis

Rations and herbs were prepared and analyzed for proximate components in accordance with the AOAC [15] and presented in Table 1. Approximately 1 gm of dried herbs and meat were placed in a separately marked crucible to determine ash at 550°C for 5 h. Then the sample was digested at 180°C using 70% HNO3 and 6 M HCl in a 3:1 ratio until completely dissolved. Then the sample was prepared, and calcium (Ca) and phosphorous (P) were measured using a spectrophotometer (T60; PG Instruments, Lutterworth, Leicestershire, UK), while zinc and iron were analyzed by adopting an atomic absorption spectrophotometer (Model no. AA-7000; Shimadzu, Kyoto, Japan). Besides, the meat (breast and thigh) sample was chopped and crushed using a mortar and pestle. Then crushed meat sample was dissolved in solution (chloroform: methanol = 2:1) and extracted total lipids as described by Folch et al. [16]. A separating funnel was used to separate the cholesterol-containing chloroform layer. Cholesterol was measured from the isolated meat cholesterol using the cholesterol kit with the help of the bio-analyzer (Urit810; URIT Medical Electronic Group Co., Ltd., Guangxi, China). The concentrations of total cholesterol (TC), triglycerides (TG), high-density lipoprotein (HDL), Ca, P, Zn, Fe, aspartate transaminase (AST), alanine amino transaminase (ALT), and alkaline phosphatase (ALP) in serum were determined using specific enzymatic kits in a Urit810 bio-analyzer. The serum low-density lipoprotein (LDL-C) and very low-density lipoprotein (VLDL-C) cholesterols were calculated by adopting the following equation of Popović et al. [17].

**Table 1.** Ingredients and nutritional composition of starter, grower diet, and herbal supplementation.

Ingredients	Amounts (%)			
	Starter diet (1–14 day)		Grower diet (15–35 day)	
Corn	47.00		50.75	
Soybean meal	39.50		35.00	
Pro-pack ^a^	4.50		4.50	
Limestone	1.10		1.10	
Di-calcium phosphate	1.80		1.50	
Soybean oil	5.30		6.35	
Lysine	0.10		0.10	
Methionine	0.10		0.10	
Broiler Premix ^b^	0.30		0.30	
Salt	0.30		0.30	
**Chemical composition,** **(% on fed basis)**	**Experimental diets**		**Supplemented herbs**	
	**Starter diet**	**Grower diet**	**Lemongrass**	**Spearmint**
Dry matter	88.53	88.25	25.09	10.80
Crude protein	22.47	20.83	5.37	13.90
Crude fiber	4.85	4.74	26.54	13.10
Ether extract	3.93	4.08	5.15	3.17
Ash	8.48	8.27	8.86	12.80
Calcium	0.95 ^c^	0.89 ^c^	0.39	2.11
Available phosphorous	0.44 ^c^	0.41 ^c^	0.09	0.005
Zinc, mg/kg	168 ^c^	149 ^c^	1,168	39
Iron, mg/kg	175 ^c^	147 ^c^	297	1,142
ME, kcal/kg ^c^	2,956	3,098	2,142	2,355

ME: Metabolizable energy.

a Pro-Pak is a protein concentrate manufactured by H. J. BAKER & BRO., LLC, USA which contained: 94.80% DM, 60% CP, 8.1% CF, 2.2% Phosphorus, 4.0% Calcium, 12.3% Ash, 2,865 kcal/kg ME, 90.0% Pepsin digestibility.

b Each kg broiler premix contained: 13.50 IU Vitamin A, 1.50 IU Vitamin D3, 50 mg a-DL-Tocopherolacetate, 2 mg Menadione, 3 mg Thiamine, 5 mg Riboflavin, 4 mg Pyridoxin, 15 pg Cobalamine, 200 pg Folsaure, 60 pg Nicotenic acid, 30 mg Ca-PLtothenate, 750 mg Cholin, 150 mg Ascorbic acid.

c Calculated value.

**Table 2.** Effects of herbal rations on production performance of broilers.

Variables	CT-R	Herbal rations			SEM	*p*-value
		LG-R	SM-R	LS-R		
FBW (gm/b)	1,496.43 ± 25.94	1,548.41 ± 22.22	1,520.24 ± 19.29	1,538.23 ± 24.47	8.235	0.100
BWG (gm/b)	1,450.56^b^ ± 24.32	1,502.15^a^ ± 20.89	1,474.11^ab^ ± 17.89	1,492.36^ab^ ± 22.84	7.964	0.078
FI (gm/b)	2,755.10^a^ ± 19.50	2,754.95^a^ ± 44.64	2,672.65^b^ ± 25.95	2,701.03^ab^ ± 32.21	13.29	0.031
FCR	1.89^a^ ± 0.03	1.83^b^ ± 0.01	1.82^b^ ± 0.01	1.81^b^ ± 0.01	0.011	0.001
PEF (%)	218.25^b^ ± 7.15	234.02^a^ ± 2.97	232.30^a^ ± 3.46	235.59^a^ ± 4.65	2.398	0.008
DY (%)	57.39^c^ ± 0.38	59.81^b^ ± 0.51	61.65^a^ ± 0.53	62.36^a^ ± 0.46	0.591	0.001

CT-R: Corn-soya based basal ration, LG-R: CT-R + 1.0% fresh lemongrass on DM basis, SM-R: CT-R + 1.0% fresh spearmint on DM basis, LS-R: CT-R + 0.5% fresh lemongrass and 0.5% fresh spearmint on DM basis, FBW: Final body weight, BWG: Body weight gain, FI: Feed intake, FCR: Feed conversion ratio, PEF: Production efficiency factor, DY: Dressing yield, gm: Gram, b: Bird, %: Percentage, SEM: Standard error mean.

^a–c ^Means values with dissimilar superscripts differ significantly (*p *< 0.05).

### Data analysis

Triplicate samples of herbs, ration, blood, and meat were tested. All data were organized in Excel for one-way analysis of variance analysis in a completely randomized design. The standard deviation was included with the mean value, and the Duncan multiple range test was used to determine the significance of the mean value. For the comparison of mean, the significance threshold was considered at 5% (*p* < 0.05). All statistical studies were conducted using Statistical Package for the Social Sciences (SPSS 11, Inc., Chicago, IL).

## Results

### Production performance of broilers

FBW of birds given herbal rations (LG-R, SM-R, and LS-R) was not affected (*p* > 0.05), while higher BWG propensity was observed in birds fed herbal rations compared to CT-R (*p* = 0.078), and the highest value was obtained in LG-R. Better FCR, PEF, and DY were observed in birds fed herbal rations than in birds offered CT-R (*p* < 0.05), while no variation was obtained among the birds offered herbal rations (*p* > 0.05). However, FI was influenced among the birds fed herbal rations and CT-R (*p* < 0.05), while the lowest FI was noted in SM-R (Table 2). 

### Lipid and mineral status of serum and meat of broilers

Birds fed herbal rations showed lower serum TG, TC, LDL, and VLDL concentrations than those fed CT-R (*p* < 0.05), but there was no notable change observed in the birds given different herbal rations (Table 3). Furthermore, as compared to broilers fed CT-R, LG-R and LS-R revealed 3.95% and 4.78% higher serum HDL concentrations, respectively (*p* < 0.05), while birds given CT-R and SM-R had no variation (*p *> 0.05). The meat cholesterol level and abdominal fat of birds fed herbal rations were lowered by 3%–5% compared to birds served CT-R (*p* < 0.05). Furthermore, substantially the highest serum Zn and Fe concentrations were observed in birds served LG-R and SM-R, respectively. In contrast, serum Ca and P concentrations in birds given different rations were consistent (*p* > 0.05). When compared to birds fed CT-R, the birds receiving LG-R and SM-R showed the highest meat Zn and Fe contents, while birds fed LS-R improved both meat Zn and Fe contents (*p* < 0.05) (Table 3). 

### Liver health and meat colors of broilers

Compared to birds given CT-R, herbal rations exhibited a propensity to enhance liver percent (*p* > 0.05). However, the concentrations of serum ALT, AST, and ALP were better in birds who offered herbal rations than in birds fed CT-R (*p* < 0.05). However, compared to birds fed CT-R, broilers given LG-R, LS-R, and SM-R had healthier serum ALT, AST, and ALP concentrations, respectively, with significant variation among birds, served herbal rations (*p* < 0.05). Furthermore, when compared to birds offered CT-R, SM-R, and LS-R, they elevated the redness (a*) and SI values of breast and thigh meat (*p* *< *0.05), but no statistical difference was observed in birds fed CT-R and LG-R (*p* > 0.05). In contrast, the lightness (L*) and yellowness (b*) of breast and thigh meat were unaffected by herbal rations and CT-R (Table 4).

## Discussions

Herbs and their extracts are gaining much attention as a replacement for AGPs in birds because they boost bird performance and product quality [17–20]. Like other herbs, lemongrass powder or oil and spearmint powder or oil used as supplements in rations accelerated broiler performance [12,21,22], which is related to this study. This improved broiler’s performance was caused by secondary metabolites in enriched herbs that showed antioxidant, anti-inflammatory, and antimicrobial activity in broilers [6,10,23]. The properties of these herbs improved normal physiological and immunological function, N-retention, and reduced muscle breakdown, as well as microbial pathogens that may trigger the release of digestive enzymes, resulting in improved nutrient digestibility, FCR, muscle mass, and overall performance in birds [4,24]. Lower FI in birds fed SM-R may be due to the presence of carvone, which causes the bitter flavor of spearmint [11,12].

**Table 3.** Effects of herbal rations on serum and meat lipid and minerals status of broilers.

Variables	CT-R	Herbal rations			SEM	*p*-value
		LG-R	SM-R	LS-R		
Serum lipid profile (mg/dl)						
TGs	141.7^a^ ± 0.66	137.2^b^ ± 0.99	139.1^b^ ± 0.94	137.5^b^ ± 1.68	0.607	0.004
TC	122.5^a^ ± 1.35	116.8^b^ ± 1.32	118.0^b^ ± 1.17	118.4^b^ ± 0.93	0.715	0.002
HDL-C	60.7^c^ ± 0.49	63.1^ab^ ± 1.00	61.9^bc^ ± 0.73	63.6^a^ ± 0.38	0.386	0.003
LDL-C	33.6^a^ ± 1.11	26.3^b^ ± 1.52	28.3^b^ ± 1.46	27.3^b^ ± 1.45	0.911	0.001
VLDL-C	28.3^a^ ± 0.13	27.4^b^ ± 0.20	27.8^b^ ± 0.19	27.5^b^ ± 0.34	0.121	0.004
Serum minerals profile						
Ca, mg/dl	8.80 ± 0.25	9.04 ± 0.10	8.91 ± 0.14	8.99 ± 0.15	0.049	0.386
P, mg/dl	5.10 ± 0.12	5.21 ± 0.15	5.13 ± 0.12	5.12 ± 0.16	0.036	0.798
Zn, µg/dl	180.4^c^ ± 0.80	187.7^a^ ± 1.76	182.1^c^ ± 0.96	184.5^b^ ± 1.05	0.887	0.001
Fe, µg/dl	122.1^b^ ± 1.11	122.5^b^1.09	126.2^a^ ± 1.78	124.6^a^ ± 0.96	0.565	0.006
Meat lipid and mineral profiles						
Abd. fat (%)	1.40^a^ ± 0.01	1.35^b^ ± 0.01	1.36^b^ ± 0.02	1.35^b^ ± 0.02	0.007	0.002
TC, mg/dl	83.33^a^ ± 0.92	79.43^b^ ± 0.90	80.26^b^ ± 0.79	80.54^b^ ± 0.63	0.485	0.002
Zn, mg/kg	9.55^c^ ± 0.013	9.60^a^ ± 0.017	9.56^bc^ ± 0.017	9.59^ab^ ± 0.016	0.007	0.015
Fe, mg/kg	6.31^c^ ± 0.014	6.32^bc^ ± 0.015	6.37^a^ ± 0.022	6.34^ab^ ± 0.028	0.008	0.012

CT-R: Corn-soya based basal ration, LG-R: CT-R + 1.0% fresh lemongrass on DM basis, SM-R: CT-R + 1.0% fresh spearmint on DM basis, LS-R: CT-R + 0.5% fresh lemongrass and 0.5% fresh spearmint on DM basis, TC: Total cholesterol, HDL-C: High-density lipoprotein cholesterol, LDL: Low-density lipoprotein cholesterol, VLDL: Very low-density lipoprotein cholesterol, Ca: Calcium, P: Phosphorous, Zn: Zinc, Fe: Iron, Abd. fat: Abdominal fat, mg/dl: Milligram per deciliter, µg/dl: Microgram per deciliter, %: Percentage, mg/kg: Milligram per kilogram, SEM: Standard error mean.

^a–c ^Means values with dissimilar superscripts differ significantly (*p* < 0.05).

**Table 4.** Effects of herbal rations on serum liver enzymes activity and meat color of broilers.

Variables	CT-R	Herbal rations			SEM	*p*-value
		LG-R	SM-R	LS-R		
Liver weight (%)	2.51^b^ ± 0.05	2.61^ab^ ± 0.04	2.59^ab^ ± 0.05	2.65^a^ ± 0.08	0.072	0.052
Serum liver enzymes activity (U/l)						
ALT	185.7^a^ ± 2.75	145.4^c^ ± 2.17	155.2^b^ ± 2.75	149.4^c^ ± 1.62	4.813	0.001
AST	4.85^a^ ± 0.33	2.70^c^ ± 0.11	3.29^b^ ± 0.38	2.91^bc^ ± 0.10	0.261	0.001
ALP	89.1^a^ ± 3.38	70.7^c^ ± 2.49	82.7^b^ ± 2.92	75.4^c^ ± 2.17	2.211	0.001
Breast meat color		
L*	56.13 ± 0.84	57.41 ± 0.68	58.30 ± 2.17	56.99 ± 1.26	0.411	0.336
a*	4.45^c^ ± 0.61	4.76^bc^ ± 0.38	5.60^a^ ± 0.39	5.33^ab^ ± 0.23	0.172	0.037
b*	11.27 ± 0.82	11.71 ± 1.13	12.00 ± 0.25	12.03 ± 0.15	0.198	0.556
SI	12.13^b^ ± 0.56	12.65^b^ ± 0.23	13.25^a^ ± 0.35	13.15^a^ ± 0.21	0.733	0.041
Thigh meat color		
L*	58.40 ± 3.89	53.29 ± 3.79	60.90 ± 3.45	58.76 ± 1.54	1.172	0.104
a*	4.66^b^ ± 0.36	4.96^ab^ ± 0.40	5.42^a^ ± 0.14	5.16^ab^ ± 0.04	0.108	0.050
b*	11.78 ± 0.57	11.90 ± 0.37	12.71 ± 0.53	12.37 ± 0.28	0.159	0.118
SI	12.67^b^ ± 0.66	12.89^ab^ ± 0.49	13.81^a^ ± 0.44	13.40^ab^ ± 0.28	0.181	0.056

CT-R: Corn-soya based basal ration, LG-R: CT-R + 1.0% fresh lemongrass on DM basis, SM-R: CT-R + 1.0% fresh spearmint on DM basis, LS-R: CT-R + 0.5% fresh lemongrass and 0.5% fresh spearmint on DM basis, ALT: Alanine aminotransferase, AST: Aspartate aminotransferase, ALP: Alkaline phosphatase, U/l: International unit per liter, L*: Lightness, a*: Redness, b*: Yellowness, SI: Saturation index, SEM: Standard error mean.

^a–c ^Means values with dissimilar superscripts differ significantly (*p* < 0.05).

In addition, both herbs are enriched with phenolic and flavonoid components that successfully decrease the concentration of serum TGs, TC, and bad cholesterol while increasing the good cholesterol and lowering the meat cholesterol in broilers, which supports the findings of previous studies [4,22,25]. Citral, a flavonoid, and carvone, a phenolic component remaining in lemongrass and spearmint herbs, respectively, have the ability to suppress 3-hydroxy-3-methylglutaryl-coenzyme A reductase activity [26], which is a basic regulating enzyme in the cholesterol production process [27]. So, decreased blood cholesterol in birds given herbal rations might be one of the prime reasons for lower cholesterol in broiler meat [22].

Furthermore, feeding bioactive component-rich herbs to birds may boost serum antioxidant status, resulting in lower reactive oxygen species, oxidative stress, peroxidation, and serum TG concentration [4,28]. This lower serum TC and TGs may suppress the fatty acid transport from the liver to adipose tissue, resulting in lower abdominal fat content as well as higher DY in the birds fed herbal rations [29], which is similar to the previous findings [20,22]. Birds served herbal rations showed a tendency toward higher liver percent due to lower dietary fat absorption [30], lipase activity [31], and/or hepatic lipogenesis [32] in broilers. Moreover, bioactive component-enriched herbs given to the bird revealed lower liver enzymatic activity, indicating less hepatic damage and a better percentage. The desired amount of citral in lemongrass and the phenolic component carvone in spearmint promoted mRNA expression and activities of CYP450 1A2, 2D22, 2E1, and 3A11 in mouse liver, resulting in reduced hepatic enzymatic activity and hepatoprotective benefits in the animals [33,34]. Higher zinc and iron content in lemongrass and spearmint, respectively, might be responsible for improving serum zinc and iron concentration in birds, which supports the previous findings [35,36].

The elevated serum zinc and iron concentrations indicated that the red meat color of birds given LG-R and SM-R, respectively, supported the previous findings [37,38]. Higher serum zinc concentrations improved the total antioxidant status of birds, which may reduce lipid peroxidation and improve meat color, whereas elevated iron in bird serum bound to hemoglobin and myoglobin, which is a key component of meat color [39,40]. The higher iron concentration in meat indicates a darker color, similar to the current study. Another reason for the improved redness of meat in birds given herbal rations seems to be that herbs are a well-known source of bioactive components that boost serum antioxidant capacity in broilers [20]. These higher levels of serum antioxidants reduced phospholipase A2 activity and lipid oxidation, which are responsible for the generation of brown metmyoglobin from bright red oxygenated myoglobin, which is associated with the red color of meat [41–43].

Moreover, improved performance and meat quality in birds may be attributed to herbal supplementation, as both herbs possess antioxidants, anti-inflammatory, antibacterial, and immune-modulating properties. Additionally, spearmint contains more iron, and lemongrass comprises more zinc, both of which have immune-modulatory effects [44], which might be examined and used to clarify the study. Also, each herb and its combination will likely increase the amount of antioxidants in blood and meat. This is something that could be studied in the future.

## Conclusion

It may be concluded that solely giving LG-R, SM-R, and LS-R enhanced the feed efficiency, production efficiency factor, and performance, DY, meat red color, and reduced the serum and meat TC level compared to CT-R. In contrast, LS-R improved bird performance, DY, and meat quality (increased meat iron, zinc, and red color, and reduced cholesterol level) by improving serum iron and zinc concentration and successfully modifying the serum lipid profiles, respectively. 

## List of abbreviation

AGPs, antibiotic growth promoters; BWG, body weight gain; CT-R, control ration; DY, dressed yield; FBW, final body weight, dl, deciliter; FCR, feed conversion ratio; FI, feed intake; IU, international unit; LG-R, lemongrass ration; LS-R, lemongrass-spearmint ration; MJ, mega-joule; ME, metabolizable energy; mg, milligram; PEF, performance efficiency factor; rpm, rotation per minute; SEM, standard error mean; SM-R, spearmint ration, wt., weight; µg, microgram; DM, dry matter; CP, crude Protein.
